# Pazopanib as a second-line treatment for non-cytokine-treated metastatic renal cell carcinoma: a meta-analysis of the effect of treatment

**DOI:** 10.1186/s12894-016-0156-4

**Published:** 2016-07-04

**Authors:** Victor C. Kok, Jung-Tsung Kuo

**Affiliations:** Division of Medical Oncology, Cancer Center of Kuang Tien General Hospital, 117 Shatien Rd, Taichung, 43303 Taiwan; Department of Biomedical Informatics, Asia University, Taichung, 41354 Taiwan; Division of Biostatistics, Institute of Public Health, School of Medicine, National Yang-Ming University, Taipei, 11221 Taiwan

**Keywords:** Pazopanib, Second-line therapy, Meta-analysis, Renal cell carcinoma, Targeted therapy

## Abstract

**Background:**

The currently recommended treatment algorithm for patients with advanced renal cell carcinoma who fail the first-line targeted therapy does not normally include pazopanib as a second-line treatment option. It would therefore be of interest to determine the efficiency of pazopanib in this setting in terms of the partial response rate (PRR), disease control rate (DCR), and progression-free survival (PFS).

**Methods:**

Peer-reviewed clinical reports without language restriction, both full papers and conference abstracts, which assessed the second-line use of pazopanib following failure of first-line non-cytokine-targeted therapy, were included. After the literature retrieval, we conducted a Preferred Reporting Items for Systematic reviews and Meta-Analyses (PRISMA)-compliant systematic review of the literature and meta-analysis of the size of the effect of each outcome measure (PRR, DCR, and PFS). The effect size and 95 % confidence interval (CI) were calculated using fixed-effect or random-effects models based on the heterogeneity represented by I^2^ of selected studies. Meta-analysis forest plots with a fixed-effect model showing the PRR and DCR were created.

**Results:**

Our results show that there are no available comparative studies on pazopanib second-line treatment. Only phase II trials or retrospective analysis reports were retrievable. Six studies (comprising 217 patients) were included in the qualitative and quantitative analysis. Pazopanib as a second-line treatment resulted in a PRR of 23 % (95 % CI, 17–31 %; I^2^ = 52.6 %) and a DCR of 73 % (95 % CI, 65–80 %; I^2^ = 0.00 %). The meta-analysis with fixed-effect model revealed that PFS was 6.5 months (95 % CI, 5.6–7.5 months; I^2^ = 86.2 %).

**Conclusions:**

In conclusion, the effectiveness and indication of pazopanib for use in the second-line setting has not yet been examined in-depth; however, this meta-analysis has shown that the treatment effects in terms of PRR, DCR, and PFS may be similar to other well-studied second-line targeted therapies. Rigorous comparative phase III trials testing this hypothesis are required.

## Background

Before the advent of targeted therapies, patients with advanced renal cell carcinoma (RCC) were treated with cytokine immunotherapy using interferon or interleukin-2. Although some long-term remission was achieved, most patients did not benefit from this treatment, but instead suffered from significant adverse effects from the immunotherapy. Health-related quality of life has been shown to be significantly poorer in patients receiving immunotherapy [1].

Up until early September 2015, at least seven targeted agents (sunitinib, sorafenib, pazopanib, axitinib, everolimus, temsirolimus, and bevacizumab) have been approved for treatment of metastatic RCC (mRCC) by the US Food and Drug Administration (FDA) [2]. In late August 2015, the FDA granted the use of lenvatinib, a multiple tyrosine kinase inhibitor, for investigational use only in patients with advanced or mRCC who had failed or did not tolerate the first-line targeted therapy [3]. Non-cytokine targeted therapy has become the first-line choice because of its favorable toxicity profiles and ease of administration in ambulatory settings. Based on limited evidence from clinical trials, expert opinions, and consensus meetings, practice guidelines on how to select these agents in sequential order and in defined risk groups have been established [4]. The rationale behind the sequential monotherapy for mRCC is mainly driven by research results from well-designed clinical trials. Treatment with combination therapeutics has been deemed to be unfeasible due to toxicity profiles, or has not been shown to be more effective than sequential monotherapy. Several population-based studies have demonstrated that sequential targeted monotherapy improves overall survival [5–7]. Nevertheless, the optimal sequencing of targeted agents to treat mRCC and maximize the patients’ survival is still under rigorous investigations [8, 9].

Pazopanib was approved by the FDA in 2009 for treatment-naïve or cytokine-failed patients with advanced RCC. Pazopanib is an oral agent that inhibits vascular endothelial growth factor (VEGF), PDGF, and C-Kit tyrosine kinase receptors. However, pazopanib as a second-line treatment for advanced RCC that is non-responsive to previous treatment with VEGF-targeted drugs has not received adequate investigation, and has never proceeded to phase III trials.

The present study involved a systematic review of the current literature with the aim of providing a more precise estimate of the treatment effects, such as the partial response rate (PRR), disease control rate (DCR), and progression-free survival (PFS) of pazopanib used in the second-line setting after a non-cytokine VEGF-targeted therapy.

## Methods

This systematic review and meta-analysis of treatment effect is reported according to the preferred reporting items for systematic reviews and meta-analyses (PRISMA) guidelines [10]. The research was performed in accordance with the Declaration of Helsinki after approval by the Kuang Tien General Hospital accredited in-house institutional review board (certificate no. KTGH-10431).

### Eligibility criteria

Peer-reviewed clinical reports without language restriction- either full papers or conference abstracts- assessing the second-line use of pazopanib after failed first-line non-cytokine-targeted therapy were included. An explicit statement of questions being addressed with reference to participants, interventions, comparisons, outcomes, and study design (PICOS) was established as follows: P- patients with metastatic or advanced RCC failing first-line non-cytokine targeted therapy; I- pazopanib as the second-line drug; C- placebo or other antineoplastic agent when regarding comparative trials; O- response rate, DCR, PFS, and overall survival (OS); S- phase III or phase II comparative or non-comparative trials and retrospective analyses would all qualify for review. Inclusion criteria were studies reporting the treatment effect in terms of response rate, DCR, PFS and/or OS, with a starting dose of pazopanib of 800 mg per day. Studies involving pure cytokine treatment prior to pazopanib treatment were excluded from the analysis.

### Search methods

Full electronic searches were performed in the PubMed biomedical literature database using medical subject headings (MeSH), a controlled vocabulary thesaurus and Boolean logic operators. The search phrase was (((“Carcinoma, Renal Cell”[Mesh]) AND pazopanib)) AND ((second-line) OR sequential OR (second line)E). Additional searches were carried out in the EMBASE database using the search terms: “pazopanib AND second AND line AND renal AND cell AND carcinoma AND ([article]/lim OR [article in press]/lim OR [conference abstract]/lim OR [conference paper]/lim OR [conference review]/lim OR [editorial]/lim OR [letter]/lim) AND [humans]/lim AND [embase]/lim.” Conference abstracts published in the annual meeting of the American Society of Clinical Oncology (ASCO) and that of the European Society for Medical Oncology (ESMO) are included in the EMBASE database. The date of the last search was 20 May 2015.

### Study selection

Duplicate records from searches of both databases were excluded. The remaining records were screened by title and abstract content for suitability of inclusion for further analysis. Although potentially confirming the existence of unpublished studies, abstracts were excluded if they did not contain enough information for this systematic review.

### Data extraction

The name of the first author, year of publication, study setting (phase II or retrospective), number of individuals in the study, proportion of male patients, median age with range, histology type of RCC, status of previous nephrectomy, 3-tier risk category such as Memorial Sloan-Kettering Cancer Center (MSKCC) risk group, Motzer, Heng, or International Metastatic RCC Database Consortium (IMDC) risk category; the first-line agent used, complete response rate, partial response rate (PRR), DCR, and PFS time were recorded. Mean age and standard deviation data were calculated when missing [11].

PFS was defined as the time from the initiation of pazopanib treatment to the date of documented progressive disease (PD) based on the Response Evaluation Criteria in Solid Tumors (RECIST) 1.0 or 1.1 criteria or death from any cause. DCR was defined as the proportion of patients who obtained an objective tumor response [complete response (CR) or partial response (PR)] or those who had achieved stable disease (SD) based on RECIST version 1.0 or version 1.1 criteria. Both older and newer versions of RECIST criteria have been widely accepted for objectively assessing responses to therapy in patients with RCC treated with targeted therapy. In a recent study investigating the concordance between RECIST 1.1 and RECIST 1.0 in patients with advanced RCC receiving VEGR-targeted therapy, Krajewski, K. M. et al. reported that response assessments were, overall, highly concordant between the two criteria and there was no evidence of a difference in time–to-progression (TTP) between the two criteria [12].

### Data synthesis and statistical analysis

#### Measuring the treatment effect

To improve the precision and the generalizability of the estimated treatment effect, data synthesis from selected studies using meta-analysis was planned. Studies were weighted before pooling. The meta-analysis forest plot using the fixed-effect model was used at the outset to calculate the rate of partial remission and DCR with 95 % confidence intervals (CI). The fixed-effect model was adopted based upon the assumption that all of the studies to be examined as a whole were considered to have been conducted under similar conditions (second-line treatment following failure of non-cytokine first-line treatment) with similar subjects (advanced/metastatic RCC); in other words, the only difference between the selected studies was their effectiveness in detecting the outcome of interest.

### Heterogeneity analysis

Heterogeneity analysis refers to the analysis of the variation in study outcomes between studies selected for the meta-analysis. Non-combinability testing for measuring heterogeneity in meta-analysis was presented using I^2^ (=100 % x (Q-df)/Q) [13]. I^2^ is an intuitive and simple expression of the inconsistency across studies and its confidence interval is constructed using the test-based method of Higgins and Thompson. An I^2^ value ≤ 24 % was considered as having minimal heterogeneity. An I^2^ value between 25 and 49 % was regarded as having low heterogeneity; an I^2^ value between 50 and 74 % had moderate heterogeneity, and an I^2^ value ≥ 75 % was regarded as having high heterogeneity. If the I^2^-value was ≥50 %, the random-effects model was used. The statistical software StatsDirect (StatsDirect Ltd, England) and Comprehensive Meta-Analysis software were used to generate the summary figures and pooled effect sizes and CI used in the meta-analysis.

## Results

**Fig. 1:**
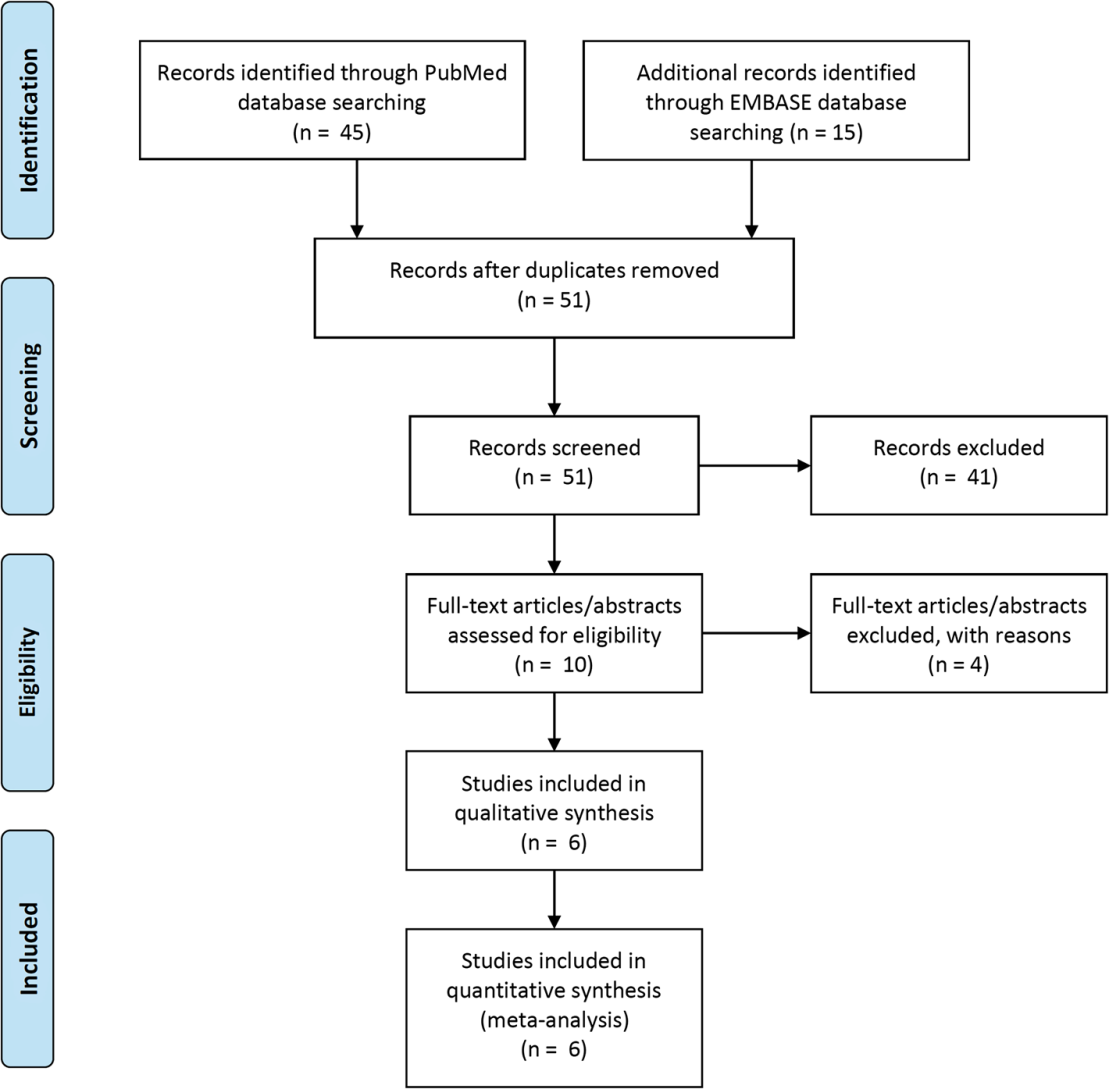
The PRISMA consort flow diagram showing the process of study selection

A total of 60 studies were identified from the PubMed (*N* = 45) and EMBASE (*N* = 15) databases. After removing duplicates, 51 retrieved records were screened according to the title and abstract for further eligibility, which subsequently excluded 41 records that did not qualify. The remaining 10 records were then assessed in detail according to the full-text with regard to eligibility for further qualitative and quantitative synthesis, which subsequently excluded four reports; thus, six studies were eventually considered to be eligible for the meta-analysis (Fig. 1). Motives for exclusion included pazopanib being used beyond second-line treatment, cytokine pretreatment, systematic review article reporting secondary results, and missing data. Figure 1 displays the PRISMA flow diagram for study selection and consists of four consecutive steps from identification in databases, screening the records, checking eligibility against the criteria for inclusion, and the final inclusion step.

There were no phase IIb or III comparative clinical trials testing pazopanib in the second-line treatment for mRCC. Six records, of which five were journal articles and one was a conference abstract, were retrieved for analysis [14–19]. All the non-comparative studies were published between 2013 and 2015. Only phase II trial data (*N* = 140), case-series, and registry data were retrievable. The total number of patients in these six studies was 217; 156 of patients, from four studies, were included for meta-analysis of the PRR, 154 from three studies for meta-analysis of the disease control rate, and 203 from five studies were included for the meta-analysis of PFS (Table 1).

**Table 1:** Selected studies pooling 205 patients for systematic review and meta-analysis and display of main patient characteristics

First author	Year publ.	Setting	*N*	Gender, % men	Age, median (range)	Clear cell type, %	Previous nephrectomy	Poor risk percent	First-line targeted therapy	PRR	DCR	PFS (months)
Xie	2015	Phase II	85	72 %	63 (41–85)	100 %	81 %	24.4 %^a^	sunitinib	15.3 % (11.2–23.9 %)	70.6 %	5.6 (4.1–6.7)
Hainsworth	2013	Phase II	55	76 %	60 (41–82)	100 %	89 %	29 %^b^	sunitinib (*N* = 39) or bevacizumab (*N* = 16)	27 % (?-?)	76 %	7.5 (5.4–9.4)
Sanchez	2013	Retrospective	32	65 %	NA	81 %	74 %	28.3 %^c^	sunitinib	NA	NA	13 (9.0–17.0)
Matrana	2013	Retrospective	17	69 %	65 (45–83)	100 %	89 %	34 % (MSKCC)	sunitinib	NA	NA	3.5 (1.0–15.5)
Rautiola	2013	Retrospective	14	61 %	65 (40–82)	90 %	90 %	19 %^c^	sunitinib	43 %	77 %	11 (4.6–15.6)
Al-Marrawi	2013	Retrospective	14	NA	50 (44–78)	92 %	90 %	17 %^c^	VEGF inhibitor^d^	0 % (0/2)^e^	NA	NA

*DCR* disease control rate, *mo* months, *MSKCC* Memorial Sloan Kettering Cancer Center risk category, *NA* data not available, *N* number of patients, *PFS* progression-free survival, *PRR* partial response rate

^a^International Metastatic Renal Cell Carcinoma Database Consortium (IMDC) prognostic model

^b^Motzer risk category

^c^Heng risk category

^d^Either one of sunitinib, sorafenib, or bevacizumab

^e^Denominator based on the availability of tumor response information

Information on patient age was retrievable in five out of the six studies (Table 1). Meta-analysis for mean age with the random-effects model [11] revealed that the mean age was 62 years (95 % CI, 60–64; range, 40–85 years). Overall, there were more male patients (range, 61–76 %). Sunitinib was used as a first-line treatment in 187 patients and bevacizumab in 16 patients. Overall response rates (CR + PR) to the first-line targeted therapy have been reported in four studies showing a rate from 16 to 33 %, with an average of 23 % [14, 15, 17, 19].

**Fig. 2:**
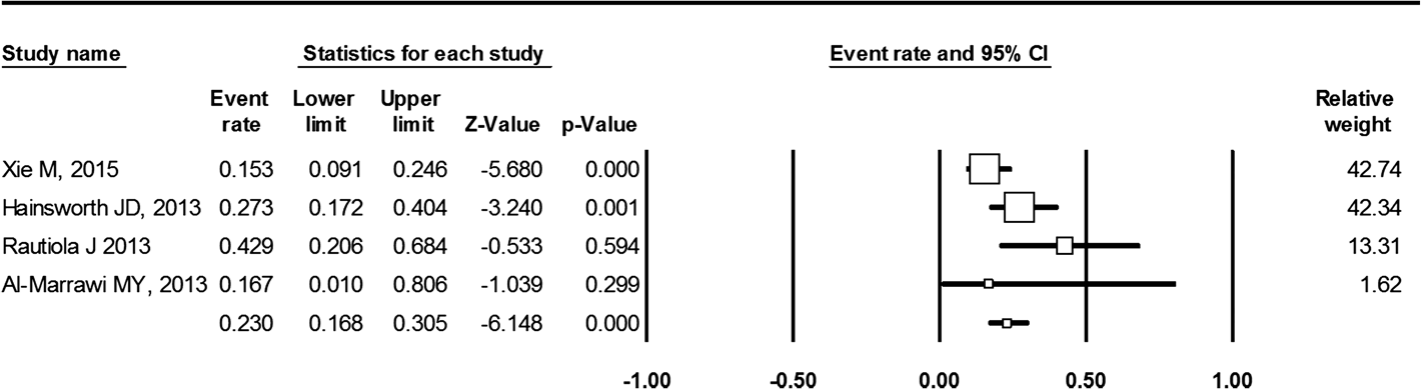
Meta-analysis forest plot with a fixed-effect model showing the partial response rate (PRR), which is 23 % with a 95 % CI of 17–31 %. The total number of individuals included in the analysis was 156. I^2^ = 52.6 %. The random-effects model estimated a PRR of 24.5 % (95 % CI, 14.5–38.4 %)

None of the patients achieved a complete response from second-line pazopanib treatment. Figure 2 displays a meta-analysis forest plot with a fixed-effect model of the PRR as the best response obtained after pazopanib used as the second-line agent. A total of four studies (comprising 156 patients) explicitly presented this rate [14, 15, 17, 19]. The reported PRR in these four studies ranged from 15.3 to 42.9 %. The fixed-effect model derived an estimated PRR of 23 % (95 % CI, 17–31 %). A heterogeneity test with I^2^ was 52.6 %, indicating the existence of a moderate degree of inconsistency across the studies. With the random-effects model, an estimated PRR would be 24.5 % (95 % CI, 14.5–38.4 %).

**Fig. 3:**
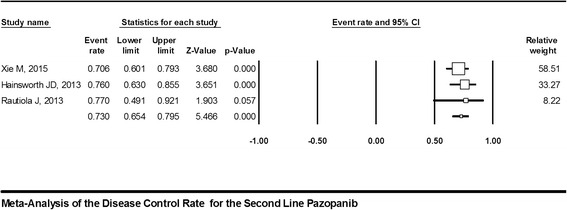
Meta-analysis forest plot with a fixed-effect model showing the disease control rate (DCR), which is 73 % with a 95 % CI of 65–80 %. The total number of individuals included in the analysis was 154. I^2^ = 0.000

Pazopanib as a second-line treatment showed a DCR of 73 % (95 % CI, 65–80 %; I^2^ = 0.00 %). Three studies reported the DCR with point estimates ranging from 70.6 to 77 % in a pool of 154 patients (Fig. 3) [15, 17, 19].

In the meta-analysis with a fixed-effect model for studies on PFS that pooled five studies- containing 203 patients- revealed a PFS of 6.3 months (95 % CI, 5.4–7.2 months; I^2^ = 83.7 %). With a relatively high degree of heterogeneity, a random-effects model was adopted, which showed a PFS of 7.6 months (95 % CI, 5.0–10.1 months) (Table 2).

**Table 2:** Meta-analysis with a fixed-effect model for pooling studies on 203 patients on progression-free survival (PFS)

Study name	Statistics for each study							
	Mean^a^	Standard error	Variance	Lower limit	Upper limit	*Z* value	*P* value	Total^b^
Rautiola, 2013	11.0	2.222	4.937	6.65	15.36	4.951	0.000	14
Matrana, 2013	3.50	1.203	1.447	1.14	5.86	2.910	0.004	17
Sanchez, 2013	13.00	1.961	3.847	9.16	16.84	6.628	0.000	32
Hainsworth, 2013	7.50	0.973	0.946	5.59	9.41	7.711	0.000	55
Xie, 2015	5.60	0.578	0.334	4.47	6.73	9.684	0.000	85
pooled synthesis	**6.29**	**0.438**	**0.192**	**5.43**	**7.15**	**14.339**	0.000	203
	I^2^ = 83.7 %							

Random-effects model estimates a median PFS of 7.6 months (95 % CI, 5.0–10.1)

^a^PFS in months; ^b^number of patients in each study; Data in bold-type are the result of data synthesis

## Discussion

Pazopanib as a second-line therapy after a non-cytokine targeted therapy in patients with mRCC has not been thoroughly evaluated. Oncologists who wish to prescribe pazopanib in the second-line setting after previous use of a targeted agent would therefore be unable to find adequate information regarding the treatment effects of pazopanib. This meta-analysis of a total of 217 patients worldwide found a PRR of 23 % (95 % CI, 17–31 %), a DCR of 73 % (95 % CI, 65–80 %), and a PFS time of 6.3 months (5.4–7.2 months).

In two recent phase III trials, axitinib therapy (5 mg twice daily), when compared with sorafenib (400 mg twice daily) as the second-line treatment in patients with metastatic clear-cell RCC, resulted in a PFS of 8.3 months (95 % CI, 6.7–9.2) in a population of globally-recruited patients (with 35 % of the patients failing to respond to first-line cytokine therapy); [20] and 6.5 months (95 % CI, 4.7–9.1) in all Asian patients (with up to 51 % of the patients failing to respond to first-line cytokine therapy) [21]. Sorafenib in these two trials gave a PFS of 5.7 months (95 % CI, 4.7–6.5) for globally-recruited patients and 4.8 months (95 % CI, 3.0–6.5) in the Asian-only population. In the Asian trial, the overall response rate was reported as 23.7 % (17–32 %), which is very similar to the current meta-analysis result [21].

PRR and PFS data for second-line non-pazopanib targeted agents following non-cytokine first-line therapy are available from the recently reported SWITCH study (only 3 % of all patients previously received cytokine treatment) [8]. The SWITCH study is a European multicenter, randomized phase III trial assessing sorafenib (first-line)-sunitinib (second-line) *vs.* sunitinib-sorafenib (i.e. in the reverse sequence) in patients with mRCC of MSKCC favorable and intermediate risk. The overall response rate in the second-line setting was 17 % in the sorafenib-sunitinib arm and 6.6 % in the sunitinib-sorafenib arm. The magnitude of the ORR is nearly halved compared to the ORR attained in the first-line targeted therapy, which was, on average 23 %, as previously mentioned. The DCR in the second-line setting regarding the sorafenib-sunitinib arm was 49 %, whereas the DCR in the sunitinib-sorafenib arm was 32 %. The PFS in the second-line setting was 5.4 months (95 % CI, 3.0–5.5 months) in the sorafenib-sunitinib sequence arm, and 2.8 months (95 % CI, 2.7–2.9 months) in the sunitinib-sorafenib sequence arm [8].

Although direct comparison of the above-mentioned studies with the current work is not recommended or correct, the study’s intention was to show the possible non-inferiority of pazopanib as a second-line therapy despite previous targeted therapy failure. Evidence-based clinical practice guidelines such as the latest 2014 European Society for Medical Oncology guidelines for diagnosis, treatment and follow-up of RCC suggest pazopanib as a second-line agent only in the setting of first-line cytokine failure [4]. Recommended second-line agents after prior targeted therapy intolerance or failure do not include pazopanib, not because of its ineffectiveness, but solely because there is not yet enough evidence available from randomized controlled trials.

What are the potential advantages when pazopanib is chosen as a second-line drug in terms of anti-neoplastic mechanisms and tolerability to prolonged use of pazopanib? Generally, pazopanib is relatively well tolerated. The most commonly reported treatment-related adverse events include diarrhea (52 %, any grade), hypertension (40 %, any grade), hair color changes (38 %, any grade), nausea (26 %, any grade), and anorexia (24 %, any grade), which tend to be fairly manageable [22]. An interesting cross-over trial demonstrated that pazopanib was superior to sunitinib in health-related quality of life (HRQoL) measures, and significantly more patients reported a preference for pazopanib over sunitinib with HRQoL and safety as the main concerns [23].

The results of the present study could be generalized to a subset of patients with mRCC whose mean age is between 60 and 64 and of the clear cell type, for whom the first-line agent was sunitinib or sorafenib. Ethnicity and prognostic risk category are irrelevant when considering generalizability. Nevertheless, in the meta-analysis, the proportion of patients with poor risk was only around 17 to 34 % representing a minority subgroup of the patients. At the time of writing, we are still lacking adequate evidence-based information on the effectiveness of each second-line targeted agent for this particular poor-risk group.

There are several notable limitations to this study. The number of studies available for the purposes of the meta-analysis was limited and mostly includes small-size studies which may lead to underpowering of the analysis. With the exception of the meta-analysis of the DCR, which had a negligible degree of heterogeneity (I^2^ = 0.000), the degree of heterogeneity of the studies was moderate when synthesizing the PRR results, and high in the meta-analysis of PFS. The two main reasons for this are that three of the selected studies had fewer than 20 patients, and four of the selected studies were retrospective analyses, while two were phase II clinical trials. The percentage of poor-risk patients in this cohort of mRCC patients was 25 %, which is similar to most of the clinical trial data with unselected patients by risk category. It was not possible to perform subgroup meta-analysis or stratify patients by risk group.

Poor-risk patients have very poor survival, with a median OS of approximately 4 months following diagnosis prior to the era of targeted therapy. With the advent of targeted therapy, a study was performed by Heng et al., in which a model of three risk categories incorporating the MSKCC model with the addition of neutrophil and platelet counts was developed and validated. In the study, the poor-risk group (*N* = 152) having 3–6 prognostic factors, had a median OS of 8.8 months and a 2-year OS of 7 % [24]. The role of pazopanib in this critical clinical scenario requires further investigations.

Currently, having no solid data such as that derived from a randomized controlled trial, pazopanib is not routinely recommended for patients with advanced or mRCC as a second-line targeted therapy after failing a prior targeted agent out of the clinical trial setting.

## Conclusion

Pazopanib as a second-line therapy for metastatic renal cell carcinoma following therapy with a targeted agent may be a viable option when other recommended drugs are less suitable for a variety of reasons, such as the patient’s inability to tolerate treatment. This meta-analysis of non-comparative studies pooling a total of 217 patients gives a more precise estimate of the treatment effects of pazopanib, such as partial response rate, disease control rate, and progression-free survival in the second-line setting.
